# Phytotoxic Effect of Invasive *Heracleum mantegazzianum* Essential Oil on Dicot and Monocot Species

**DOI:** 10.3390/molecules24030425

**Published:** 2019-01-24

**Authors:** Martina Matoušková, Jana Jurová, Daniela Gruľová, Anna Wajs-Bonikowska, Marek Renčo, Vincent Sedlák, Janka Poráčová, Zuzana Gogaľová, Danuta Kalemba

**Affiliations:** 1 Institute of Parasitology, Slovak Academy of Sciences, Hlinkova 3, 04001 Košice, Slovakia; matouskova@saske.sk (M.M.); jjurova@saske.sk (J.J.); renco@saske.sk (M.R.); 2 Department of Ecology, Faculty of Humanities and Natural Sciences, University of Prešov, 17. Novembra 1, 08001 Prešov, Slovakia; 3 Institute of General Food Chemistry, Lodz University of Technology, Stefanowskiego 4/10 St., 90924 Łódź, Poland; anna.wajs@p.lodz.pl (A.W.-B.); danuta.kalemba@p.lodz.pl (D.K.); 4 Department of Biology, Faculty of Humanities and Natural Sciences, University of Prešov, 17. Novembra 1, 08001 Prešov, Slovakia; vincent.sedlak@unipo.sk (V.S.); janka.poracova@unipo.sk (J.P.); zuzana.gogalova@smail.unipo.sk (Z.G.)

**Keywords:** allelopathy, biological activity, GC-MS, giant hogweed, aliphatic esters

## Abstract

Spreading of the plant species in new areas is supported by the hypothesis in which chemicals produced by alien species are allopathic to native plants. A novel weapon hypothesis was tested by using essential oil of dangerous alien species *Heracleum mantegazzianum* in laboratory conditions. Aboveground plant material was collected in south-east part of Slovakia, dried and hydrodistilled for essential oil isolation. Dominant compounds as octyl acetate (62.6%), hexyl 2-metylbutyrate (10.7%), hexyl isobutyrate (7.5%) and hexyl butyrate (6.5%) were identified by GC-MS. Potential phytotoxic activity was tested on three dicot plant species garden cress (*Lepidium sativum* L.), radish (*Raphanus sativus* L.) and lettuce (*Lactuca sativa* L.) and on one monocot plant species wheat *Triticum aestivum* L. Germination of the seeds of model plant species after influencing by different doses of essential oil of *H. mantegazzianum* as well as the roots length was evaluated. *Lepidium sativum* L. and *Raphanus sativus* L. were generally not sensitive to applied doses of essential oil although a little stimulation effect at some concentrations prevailed over inhibition effect. Similarly, in monocot species *Triticum aestivum* L., stimulation was visible in both root length and root number at two or one highest doses, respectively.

## 1. Introduction

*Heracleum mantegazzianum* Sommier et Levier (giant hogweed) belongs to the genus Apiaceae. It was introduced to Europe from central Asia (Caucasus) as an ornamental species. The first information about its presence in Europe comes from London`s botanical garden Kew Gardens in 1817 [1,2]. Nowadays, the plant is considered to be extremely dangerous in Slovakia as well as in other European countries as Czech Republic, Germany, Poland, Switzerland, and countries of Benelux, Great Britain etc. [3,4,5,6,7].

Giant hogweed is a plant of impressive growth. In the conditions characteristic for central Europe, it grows from 2 until 5 m. In original ecosystem it grows only until 1.5 m high. Plant is monocarpic [1], which means that under natural conditions, it dies after fructification [8]. Full bloom starts from middle June and last until the end of July. The species produce 20,000–30,000 seeds, in extreme occasion 100,000 seeds per plant individual. In addition, it is found mainly on anthropogenic sites, most often along roads and waterways. They also spread to neglected meadows, forest clearings, and places around parks. Since it is a species with strong vitality, rapid growth, large seed production and lack of natural enemies, its spreading to new sites is uncontrollable by common methods.

Chemicals produced by alien species are allopathic to native plants. This is also called as a ‘novel weapon hypothesis’ [9]. This effect spreads alien species to new areas and suppress original flora [6]. Mentioned hypothesis was already tested on invasive species such as *Eucalyptus globulus* [10], *Plantago virginica* [11], *Solidago gigantea*, *Impatiens glandulifera*, *Erigeron annuus* [12,13] and other.

Giant hogweed produces a large amount of chemical compounds. The novel weapon hypothesis can be helpful to understand the success of the giant hogweed in Europe [14]. Few studies were performed to evaluate impact of water extracts and root exudates of giant hogweed to native species [6,15,16,17]. Furanocoumarins produced by giant hogweed have negative effects on the germination of native species affected by soil or soil water extracts [14]. The water extract from the aboveground parts of giant hogweed were tested as inhibitors of the seed germination [2] and seedling growth (shoot length, root length) [6].

Volatiles as essential oil (EO) present second class of abundant compounds in genus *Heracleum* [18]. Composition of essential oil isolated by hydrodistillation from giant hogweed seeds as well as their potential antimicrobial effect was previously determined [7,18,19].

Thousands of plants belonging to different families are known to produce volatile oils serving as pollinator attractants, determinants of vegetation patterning or regulator of community structure via allelopathy [20].

Present study evaluates the content and composition of EO hydrodistilled from giant hogweed as well as the impact of EO on monocot (wheat—*Triticum aestivum* L.) and dicot species (garden cress—*Lepidium sativum* L., radish—*Raphanus sativus* L. and lettuce—*Lactuca sativa* L.). According to our knowledge this is the first report about the potential phytotoxic effect of EO from giant hogweed.

## 2. Results

### 2.1. Essential Oil Content and Composition

Total amount of EO hydrodistilled from the dried samples of giant hogweed collected on July was 0.91% of dry mass. There were identified 35 components which constituted 98.0% of total compounds. Dominant components were octyl acetate (62.6%), hexyl 2-methylburate (10.7%), hexyl isobutyrate (7.5%) and hexyl butyrate (6.5%). Four other components (octanal, hexyl acetate, hexyl hexanoate and octyl 2-methylbutyrate) were in the amounts slightly over 1% (Table 1). The rest of identified components were in the amounts below 1%.

### 2.2. Phytotoxic Effect

The biological effect of EO hydrodistilled from giant hogweed toward three dicot species (*Lepidium sativum* L., *Raphanus sativus* L. and *Lactuca sativa* L.) and one monocot species (*Triticum aestivum* L.) was evaluated as (a) influence on seed germination of four species (Table 2), (b) root elongation of four species and (c) number of roots in *Triticum aestivum*, respectively (Table 3). Six different doses (from 0.062 to 2.5 μg/mL) of EO were applied on seeds and their effect was evaluated.

Evaluation of seed germination was based on number of germinated seeds from 10 seeds placed into the Petri dish in the beginning of experiment (Table 2). Each EO dose was triplicated. No statistical difference was noted for evaluation seeds of *L. sativum* and *R. sativus* that appeared resistant toward applied concentrations of giant hogweed EO. On the contrary, *L. sativa* was sensitive to all EO concentrations. All ten seeds were germinated in control Petri dish, while only a slight difference was noted between control and the highest (2.5 μg/mL) and two lower doses (0.25 and 0.125 μg/mL). The number of germinated seeds in mentioned doses decreased from 8.7 ± 0.5 to 7.0 ± 0.8. The highest impact was noted in lowest dose (0.062 μg/mL) where the number of germinated seeds was about 47% lower (5.3 ± 1.3 germinated seeds) comparing to control. Impact of EO on germination of monocotyledonous seeds of *T. aestivum* was similar to that of lettuce. Statistical significance was noted in three doses (1.25, 0.25 and 0.062 μg/mL) and numbers of germinated seeds were 8.0 ± 0.0, 6.7 ± 0.9 and 6.3 ± 0.9, respectively.

More variable effect was noted in evaluation of root elongation after application of EO in different doses (Table 3). Except expected inhibition effect, also stimulation effect was noted. Significant stimulation effects were observed in dicotyledonous model plants. Roots of *L. sativum* at the lowest EO dose (0.062 μg/mL) were 10% longer in comparison to control. In monocotyledonous model plant *T. aestivum* the influence was the most significant at the highest dose (2.5 μg/mL) when the roots were 67% longer than in control.

On the contrary, inhibitory effect on root elongation was noted in *L. sativa* at all EO doses although only at three doses differences were statistically significant. Specific evaluation focused on different number of roots in monocotyledonous plant species. The average number in control was 2.73 roots. Significant difference was evaluated after application of the highest dose (2.5 μg/mL) when average number of roots increased to 3.5 roots (*p* = 0.05).

## 3. Discussion

The content of EO isolated from giant hogweed was previously determined for different parts of the plant. There were noted 0.45% in stems and 0.95% in fruits [19], as well as others evaluated 3% of EO in fruits [18]. Our finding of 0.91% EO in whole aerial parts is within the range presented by other authors.

Comparison of the identified compounds in giant hogweed EO also revealed good accordance with published study. As a major component octyl acetate was previously identified followed by hexyl-2-methylbutyrate, octanol, octyl butyrate, octyl-2-methylbutyrate, hexyl acetate, octyl isobutyrate and hexyl isobutyrate in flowers and fruits, p-cymene in roots, and stems and β-guaiene in leaves [18,19]. Aliphatic esters were identified as the main group of compounds in EO of giant hogweed from Poland [7]. Dominated components in EO identified in giant hogweed from Russia were octyl butyrate (32.0%), octyl acetate (18.0%) and hexyl butyrate (9.2%) [23]. Similar composition of seed EO as in *H. mantegazzianum* was noted in *H. sosnowskyi* [24]. Chemical composition of *H. mategazzianum* as well as *H. sosnowskyi* seed EOs that contained mainly octanol and hexanol esters of acetic acid, butyric and isobutyric acids differ from most of the evaluated EOs, in which terpenes dominate.

The root elongation response of *L. sativum* on EO could be generally explained by the hormesis theory. Hormesis can be defined as a biphasic response in which high doses of a toxic agent could cause inhibition of growth while low dose of the same toxic can cause stimulation [25]. The factors responsible for inducing hormesis are known as hormetins or stressors. In this sense, it has been established that in plants the challenge with different levels of stress constitutes an adaptive process, having reminiscence with the phenomenon of hormesis abovementioned. This stress can be established as “eustress” (beneficial stress) if the effect is similar to the hormetic effect in low doses of a toxin, or “distress” (harmful stress) if the level of this generates an irreversible or negative damage in the plant [26]. This explanation could be applied to the different effects visible in *Lactuca sativa* and *Triticum aestivum* in evaluation of germination and root elongation.

No significant differences were found in the chemical composition of the examined seed samples of *Heracleum sosnowskyi* and *Heracleum mantegazzianum* (Apiaceae), which confirms the suggestions that the species can be closely related [7]. The study useful for comparison of our results tested allopathic effect of EO isolated from *H. sosnowskyi* seeds on two dicots and four monocot species [24]. Different susceptibilities to *H. sosnowskyi* EO was evaluated in seeds germination as well as in radical length. The most susceptible were *Bromus secalinus* and *Avena fatua* (both monocots). Other comparable study was done by cotton swab method to characterize the effect of volatiles from *H. sosnowskyi* fruits on *Lactuca sativa*. The radicle and hypocotyl growth of lettuce seedlings were significantly inhibited while the germination of lettuce seeds was not affected by volatiles from the *H. sosnowskyi* fruits [27].

The chemical composition of essential oil varies greatly among species. While aliphatic esters dominate in *Heracleum* species, the compositions of the majority of known EOs generally contain terpenes. Many of the species in which terpenes dominated have been tested for potential phytotoxic effect [28,29,30,31].

Some scientific study of phytotoxic activity of EO reflects the different impact on monocot and dicot plant species. Different studies reported that monocot species are more resistant to EOs from plants belonging to different families (Cupressaceae, Asteraceae, Anacardiaceae, Cardiopteridaceae, Lamiaceae, Polygonaceae, Rutaceae) comparing to dicot species [28,32,33]. On the other hand, evaluation of phytotoxic activity of *S. terrae-albae* (Asteraceae) EO on a dicot plant *Amaranthus retroflexus* and a monocot plant *Poa annua* revealed that the monocot plant, *P. annua*, was more sensitive to essential oil than dicot one [34].

## 4. Materials and Methods

### 4.1. Plant Material

Plant material was collected during the vegetative season 2017 at Lekárovce (GPS 48.608129 E 22.140227) in the eastern part of Slovakia. Collection was done in July when the plant was fully maturated. There were collected aboveground parts (whole stems with leaves, flowers and seeds) from different individuals which presented fresh plant material. Plant material was placed on thin layer on filter paper and was left in room temperature approximately 14 days, until all plant material was possible to crumble.

### 4.2. Essential Oil Isolation

The dried plant material were cut into small pieces and divided into three proportionally similar samples. Then plant material was subjected into the flasks to hydrodistillation in a Clevenger-type apparatus (Kavaliergalss, Sázava, Czech Rep.). Distillation lasted 3 h. Isolated EO was placed in dark vials at +4 °C until other analysis. The plant material yielded pale-yellow oils with strong floral odor.

### 4.3. Chromatographic Analysis

Essential oil was analyzed by GC-MS-FID. The analysis was performed on a Trace GC Ultra coupled with DSQII mass spectrometer (Thermo Electron, Waltham, MA, USA). A simultaneous GC-FID and MS analysis was performed using a MS-FID splitter (SGE Analytical Science, Ringwood Victoria, Australia). Mass range was 33–550 amu, ion source-heating: 200 °C, ionization energy: 70 eV. One microliter of essential oil solution (80% *v/v*) diluted in pentane: diethyl ether was injected in split mode at split ratios (50:1). Operating conditions for capillary column Rtx-1 MS (60 m × 0.25 mm i.d., film thickness 0.25 μm), and temperature program: 50 (3 min)–300 °C (30 min) at 4 °C/min. Injector and detector temperatures were 280 °C and 300 °C, respectively. Carrier gas was helium (constant pressure: 300 kPa).

### 4.4. Identification of Compounds

The identification of compounds was based on a comparison of their mass spectra (MS) and linear retention indices (RIs, non-polar column), determined with reference to a series of alkanes C8–C24, by comparing with those in MassFinder 3 [21], Adams [22] as well as with computer mass libraries NIST 2012 and the Wiley Registry of Mass Spectral Data 8th edition.

### 4.5. Model Plants

Evaluation of potential phytotoxic effect of EO was evaluated on seeds of four species. There were chosen three dicotyledonous species as *Raphanus sativus* L. (radish), *Lepidium sativum* L. (garden cress) and *Lactuca sativa* L. (lettuce) and one monocotyledonous species *Triticum aestivum* L. (common wheat) as a model plants. *R. sativus* L. var. radicula Pers. (cv. ’Duo’), *L. sativum* L. (cv. ’Dánska’), and *L. sativa* (cv. Král Máje I.) seeds were purchased from Zel Seed (Slovakia). Common wheat was obtained from the Research Center in Malý Šariš.

### 4.6. Phytotoxic Activity Assay

Phytotoxic assay followed previously used method [35]. Two factors were taken into account in the experimental treatment: (i) four test plants: [radish (*R. sativus* L.), garden cress (*L. sativum* L.), lettuce (*L. sativa* L.) and common wheat (*T.aestivum* L.)] and (ii) six different *H. mantegazzianum* essential oil concentrations: (2.5, 1.25, 0.625, 0.25, 0.125, and 0.062 μg/mL). The essential oils were dissolved in distilled water/acetone 99.5:0.5 and diluted to prepare the desired concentrations. Distilled water/acetone 99.5:0.5 was used as control. Test seeds were surface sterilized in 95% EtOH for 15 s and rinsed thrice in distilled water. Ten sterilized seeds were sown into each Petri dish (90 mm dia) containing 5 layers of Whatman filter paper. In each Petri dish 7 mL of essential oil solutions of different concentration or distilled water/acetone 99.5:0.5 was added. Each treatment was triplicated. The Petri dishes were kept in growth chamber (20 ± 1 °C, natural photoperiod, Sanyo, MLR-351H). Evaluation of germination and the radicle length (cm) was measured after 120 h.

### 4.7. Statistical Analysis

Data from the experiment were subjected to analysis of variance (ANOVA) by Least Significant Difference’s Test. Statistical analyses were performed using the PlotIT ver. 3.2 program (Scientific Programming Enterpises, Haslett, MI, USA).

## 5. Conclusions

The aims of the study were based on the novel weapon hypothesis. The present study evaluated phytotoxicity of volatiles produced by alien species *H. mantegazzianum* to model plants. According to our findings, we can conclude that EO hydrodistilled from the alien plant species presented biological activity on the dicot and monocot plant species. The most sensitive was *Lactuca sativa* comparing to *Lepidium sativum* and *Raphanus sativus* in seed germination as well as in root length evaluation. Stimulation effect was visible in both root length and root number at two or one highest doses, respectively in monocot species. *Triticum aestivum* L.

Composition of EO is characteristic for the genus *Heracleum* where the dominant compounds are aliphatic esters which differ from dominant terpenoids in different plant groups. Regardless the EO composition, the volatile components presented similar biological effect.

## Figures and Tables

**Table 1:** Content of components in *Heracleum mantegazzianum* essential oil.

Compound Name	[%]	RI	RI Lit.
*n*-Nonane	0.1	899	900
α-Pinene	t	927	934
Isobutyl butyrate	0.1	937	939
Octanal	1.3	979	982
*n*-Hexyl acetate	1.1	993	995
*p*-Cymene	0.5	1012	1016
*n-*Butyl 2-methylbutyrate	0.2	1024	1026
(E)-β-Ocimene	t	1036	1042
3-Methylbutyl butyrate	t	1039	1041
γ-Terpinene	0.1	1049	1055
Octan-1-ol	0.5	1059	1063
Terpinolene	0.1	1074	1081
*n-*Hexyl propionate	0.4	1084	1085
*n-*Hexyl isobutyrate	7.5	1133	1132
*n-*Hexyl butyrate	6.5	1173	1176
*n-*Octyl acetate	62.6	1200	1191
(Z)-Oct-3-enyl acetate	0.3	1208	1200
*n-*Hexyl 2-methylbutyrate	10.7	1228	1224
*n-*Hexyl isovalerate	0.8	1229	1227
Lavandulyl acetate	0.1	1268	1270
Octyl propionate	0.1	1284	1280
*n-*Octyl isobutyrate	0.8	1327	1329
*n-*Hexyl hexanoate	1.4	1367	1371
*n-*Octyl butyrate	0.4	1371	1371
Dec-9-enyl acetate	0.1	1374	1378
β-Bourbonene	0.1	1379	1378
*n-*Decyl acetate	0.1	1390	1390
(*E*)-β-Caryophyllene	0.1	1413	1418
*n-*Octyl 2-methylbutyrate	1.1	1416	1421
Germacrene D	0.3	1476	1479
δ-Cadinene	t	1514	1520
*n-*Octyl hexanoate	0.5	1558	1567
β-Caryophyllene oxide	0.1	1570	1578

t—traces less than 0.05%; RI—retention index compared between software prediction and literature [21,22].

**Table 2:** Effect of different doses of the essential oil from aerial parts of *Heracleum mantegazzianum* on the seed germination of four model plant species.

	Number of Germinated Seeds ± SD			
EO Dose [μg/mL]	*Lepidium sativum*	*Raphanus sativus*	*Lactuca sativa*	*Triticum aestivum*
control	10.0 ± 0.0 ^a^	10.0 ± 0.0 ^a^	10.0 ± 0.0 ^a^	9.0 ± 1.0 ^a^
0.065	10.0 ± 0.0 ^a^	10.0 ± 0.0 ^a^	5.3 ± 1.3 ^c^	6.3 ± 0.9 ^b^
0.125	10.0 ± 0.0 ^a^	9.7 ± 0.5 ^a^	7.0 ± 0.8 ^b^	8.7 ± 0.9 ^a^
0.250	10.0 ± 0.0 ^a^	10.0 ± 0.0 ^a^	7.0 ± 1.4 ^b^	6.7 ± 0.9 ^b^
0.625	10.0 ± 0.0 ^a^	9.3 ± 0.5 ^a^	9.8 ± 0.8 ^a^	8.3 ± 0.5 ^a^
1.250	10.0 ± 0.0 ^a^	10.0 ± 0.0 ^a^	8.0 ± 0.8 ^a^	8.0 ± 0.0 ^ab^
2.500	10.0 ± 0.0 ^a^	9.3 ± 0.5 ^a^	8.7 ± 0.5 ^ab^	9.3 ± 0.5 ^a^

Each value is an average of 3 replications; SD = standard deviation; the letters a,b,c in each column present statistical differences according to Least Significant Difference’s Test (*p* = 0.05).

**Table 3:** Effect of different doses of the essential oil from aerial parts of *Heracleum mantegazzianum* on the root elongation of four model plant species and roots number of monocotyledoneous species.

	Radical Elongation [cm]				Roots Number
EO Dose [μg/mL]	*Lepidium sativum*	*Raphanus sativus*	*Lactuca sativa*	*Triticum aestivum*	*Triticum aestivum*
control	10.69 ^a^	4.52 ^a^	1.58 ^a^	3.13 ^a^	2.73 ^a^
0.065	11.82 ^b^	4.35 ^a^	0.55 ^c^	2.74 ^a^	2.53 ^a^
0.125	10.94 ^a^	4.11 ^a^	0.86 ^b,c^	2.69 ^a^	2.90 ^a^
0.250	10.12 ^a^	5.04 ^a^	1.28 ^a,b^	2.00 ^b^	2.36 ^a^
0.625	11.11 ^a^	4.98 ^a^	1.15 ^a,b^	2.19 ^a^	2.70 ^a^
1.250	9.30 ^c^	4.88 ^a^	0.99 ^b,c^	3.21 ^a^	2.63 ^a^
2.500	11.28 ^a^	4.52 ^a^	1.38 ^a,b^	5.22 ^c^	3.50 ^b^

Each value is an average of 3 replications; the letters a,b,c in each column present statistical differences according to Least Significant Difference’s Test (*p* = 0.05).
